# Case Report: The leopard sign as a potential characteristic of chronic granulomatous disease-associated colitis, unrelated to colitis severity

**DOI:** 10.3389/fimmu.2023.1208590

**Published:** 2023-12-13

**Authors:** Takuro Nishikawa, Takahiro Tomoda, Aki Nakamura, Jun Nagahama, Akihito Tanaka, Shuji Kanmura, Mari Kirishima, Akihide Tanimoto, Tsubasa Okano, Takahiro Kamiya, Kentaro Okamoto, Susumu Kirimura, Tomohiro Morio, Yasuhiro Okamoto, Hirokazu Kanegane

**Affiliations:** ^1^ Department of Pediatrics, Graduate School of Medical and Dental Sciences, Kagoshima University, Kagoshima, Japan; ^2^ Department of Pediatrics and Developmental Biology, Graduate School of Medical and Dental Sciences, Tokyo Medical and Dental University (TMDU), Tokyo, Japan; ^3^ Digestive and Lifestyle Diseases, Kagoshima University Graduate School of Medical and Dental Sciences, Kagoshima, Japan; ^4^ Department of Pathology, Graduate School of Medical and Dental Sciences, Kagoshima University, Kagoshima, Japan; ^5^ Department of Pediatric Surgery, Graduate School of Medical and Dental Sciences, Tokyo Medical and Dental University (TMDU), Tokyo, Japan; ^6^ Department of Pathology, Graduate School of Medical and Dental Sciences, Tokyo Medical and Dental University (TMDU), Tokyo, Japan; ^7^ Department of Child Health and Development, Graduate School of Medical and Dental Sciences, Tokyo Medical and Dental University (TMDU), Tokyo, Japan

**Keywords:** chronic granulomatous disease, colitis, endoscopy, haematopoietic cell transplantation, leopard sign

## Abstract

**Background:**

Chronic granulomatous disease (CGD) is an inborn immune disorder in which the phagocytic system cannot eradicate pathogens, and autoinflammation occurs. Approximately half of the patients have associated gastrointestinal symptoms. Although most cases with CGD-associated colitis present nonspecific histology, colonoscopy in some cases shows brownish dots over a yellowish oedematous mucosa, which is termed a “leopard sign”. However, the significance of these signs remains unclear.

**Methods:**

We collected data from patients with CGD whose colonoscopic findings showed the leopard sign.

**Results:**

Three patients with CGD and leopard signs were enrolled in this study. One patient underwent colonoscopy for frequent diarrhoea and weight gain failure, and another for anal fistula. The third patient was without gastrointestinal symptoms and underwent colonoscopy as a screening test before allogeneic haematopoietic cell transplantation (HCT). Endoscopic findings showed a mild leopard sign in the first case; however, non-contiguous and diffuse aphthae were observed throughout the colon. The other two cases were unremarkable except for the leopard sign. All the patients achieved remission with oral prednisolone or HCT. One patient underwent colonoscopy after HCT; results revealed improvements in endoscopy (including the leopard sign) and histological findings. However, another patient underwent colonoscopy after prednisolone treatment; this revealed no change in the leopard sign.

**Conclusion:**

The leopard sign in the colon may be a characteristic endoscopic finding of CGD, even in patients who do not develop severe gastrointestinal symptoms; however, it does not reflect the severity of CGD-associated colitis.

## 1 Introduction

Chronic granulomatous disease (CGD) is a life-threatening inborn error of immunity that is caused by pathogenic variants in the genes encoding the nicotinamide adenine dinucleotide phosphate (NADPH) oxidase complex. Hemizygous variants in *CYBB* cause X-linked CGD, whereas biallelic pathogenic variants of *CYBA*, *NCF1*, *NCF2*, *NCF4*, and *CYBC1* cause autosomal recessive forms. CGD is characterised by recurrent bacterial and fungal infections and inflammatory complications (1, 2). A typical inflammatory complication of the gastrointestinal (GI) tract is CGD-associated colitis, which causes abdominal pain, abdominal distention, diarrhoea, bloody stool, and tenesmus. This form of colitis can occur in up to 50% of patients (3). Moreover, chronic inflammation causes anaemia, thrombocytosis, elevated C-reactive protein, faecal calprotectin (FCP), hypoalbuminaemia, and failure to thrive. Endoscopy reveals diffuse colitis, patches, friability, aphtha, ulcers, and pseudopolyps. The overall disease distribution typically resembles that of colitis. In contrast, the histological findings resemble those of Crohn’s disease, which is characterised by granuloma, cryptitis, crypt abscesses, a predominance of eosinophils, and a paucity of neutrophils (4–6). Although these endoscopic and pathological findings are nonspecific, Obayashi et al. (7) reported specific endoscopic findings in three Japanese patients with CGD-associated colitis. These findings were characterised by the appearance of brown dots distributed across the yellowish oedematous mucosa and were termed “leopard sign”. Moreover, a Belgian patient with CGD also presented with a “leopard sign” in the colon (8). However, the significance of these signs remains unclear.

We conducted endoscopy on four out of seven patients diagnosed with CGD and identified the leopard sign in three of them. Herein, we report three Japanese patients with CGD who presented with the leopard sign, providing a description of the clinical significance associated with these characteristic endoscopic findings in CGD-associated colitis.

## 2 Case description

Informed consent was obtained from the parents of all patients. The study was conducted in accordance with the Helsinki Declaration and approved by the Ethics Committee on Clinical Research, Sakuragaoka Campus, Kagoshima University, and the Ethics Boards of Tokyo Medical and Dental University.

Patient 1 was a 1-year-old male with an unremarkable family history. The patient was referred to Kagoshima University Hospital with refractory cervical and axillary lymphadenitis.

**Figure 1:**
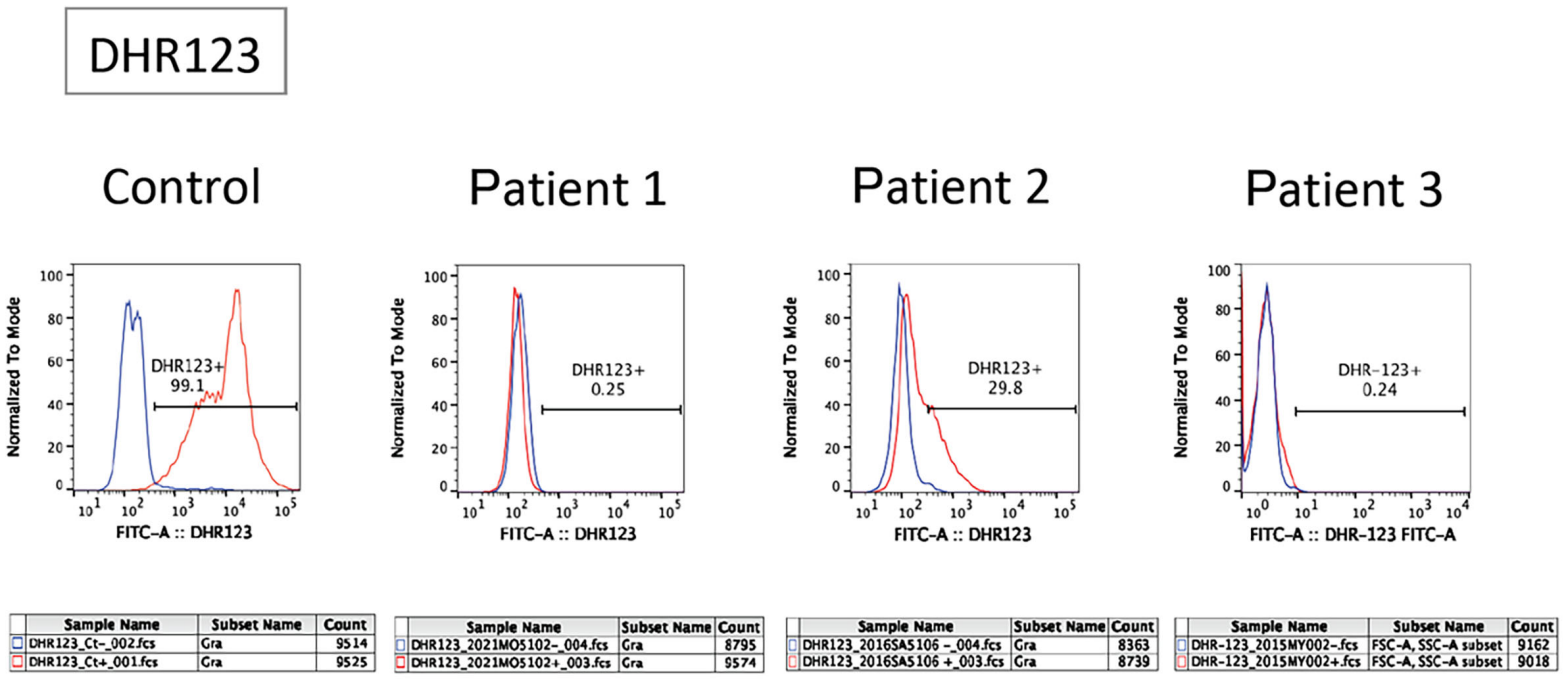
Flow cytometric analysis of dihydrorhodamine (DHR) oxidation and expression of gp91*^phox^* in patients. The panel shows a flow cytometric analysis of DHR123 in neutrophils. Blue and red lines indicate unstimulated and phorbol myristate acetate-stimulated neutrophils, respectively.

A flow cytometry-based dihydrorhodamine (DHR) 123 test using phorbol myristate acetate (PMA; 100 ng/mL) as a stimulant (9) showed no shift in neutrophils after stimulation (**Figure 1**). Next-generation sequencing (NGS) using a targeted CGD gene panel revealed a hemizygous nonsense variant (c.1011G>A, p.Trp337Ter) in exon 9 of *CYBB*. He was diagnosed with X-CGD, and therapy was initiated with trimethoprim-sulfamethoxazole (TMP/SMX) and itraconazole as prophylaxis. Three months after the diagnosis, he experienced frequent diarrhoea and weight gain failure; therefore, he underwent colonoscopy. The FCP level was markedly elevated at 1,830 mg/kg (normal value: < 50 mg/kg).

Patient 2 was a 6-year-old male with an unremarkable family history. The patient was referred to Kagoshima University Hospital with a refractory perianal abscess. The DHR123 flow cytometry assay demonstrated substantially low neutrophil oxidative burst activity (**Figure 1**). Using a targeted CGD gene panel, NGS revealed a hemizygous missense variant (c.544A>T, p.Ile182Phe) in exon 6 of *CYBB*. The patient was diagnosed with X-CGD, and therapy was initiated with TMP/SMX and itraconazole as prophylaxis. Three months after the diagnosis, although he had no GI symptoms, he had a history of an anal fistula and did not gain weight within a year. Consequently, he underwent colonoscopy, revealing an FCP level of 72 mg/kg.

Patient 3 was a 4-year-old male who was referred to the Tokyo Medical and Dental University Hospital with refractory cervical lymphadenitis at the age of 4 months. Regarding family history, the patient had a cousin with X-CGD. The DHR123 flow cytometry assay demonstrated deficient neutrophil oxidative burst activity (**Figure 1**). The patient was diagnosed with X-CGD using targeted sequencing of *CYBB* (c.1022C*>*T, p.Thr341Ile, a hemizygous missense variant in exon 9). Prophylactic therapy with TMP/SMX and itraconazole was initiated; however, the patient developed pulmonary aspergillosis. Consequently, treatment was switched from itraconazole to voriconazole. Therefore, the patient was scheduled to undergo allogeneic haematopoietic cell transplantation (HCT) for refractory aspergillosis. Although the patient had no severe GI symptoms, a colonoscopy was performed as a screening test before HCT. The characteristics of the three cases are summarised in the table (**Table 1**).

**Table 1:** Patient characteristics.

	Patient 1	Patient 2	Patient 3
Age at diagnosis of CGD	1 y	6 y	4 m
Family history	No	No	Yes (cousin)
Symptom at diagnosis	Lymphadenitis	Perianal abscess	Lymphadenitis
*CYBB* mutation	W337*	I182F	T341I
Growth failure	Present	Present	None
Prophylactic treatment	TMP/SMX, itraconazole	TMP/SMX, itraconazole	TMP/SMX, itraconazole, voriconazole
Symptoms at the time of colonoscopy	Diarrhoea	Anal fistula	None
FCP level at the time of colonoscopy	1,830 mg/kg	79 mg/kg	N.D.
Age at endoscopy	1 y 3m	6 y	4 y
Treatment for colitis	Prednisolone	Prednisolone	None
HCT	No	No	Yes
Response to treatment	Remission	Remission	Remission

M, months; Y, years; CGD, chronic granulomatous disease; TMP/SMX, trimethoprim-sulfamethoxazole; FCP, faecal calprotectin; N.D., no date; HCT, haematopoietic cell transplantation.

### 2.1 Endoscopic findings

**Figure 2:**
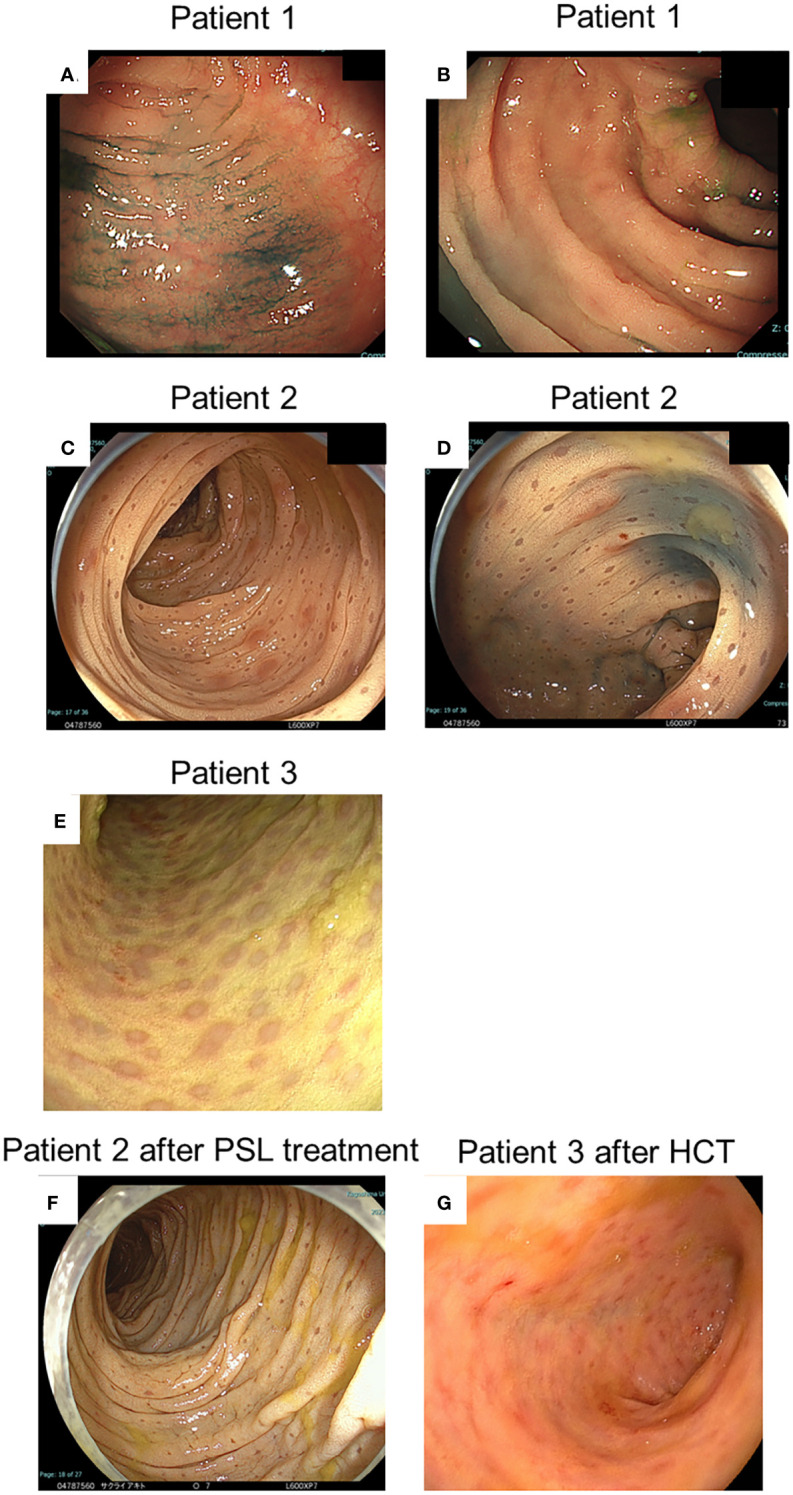
Endoscopic findings at diagnosis and after treatment of patients. **(A, B)** Indicate the endoscopic findings of patient 1; **(C, D, F)** Indicate those of patient 2; and **(E, G)** Indicate those of patient 3, respectively. **(A-E)** Indicate findings at diagnosis. **(F)** Indicates findings after prednisolone (PSL) treatment. **(G)** Indicates findings after haematopoietic cell transplantation (HCT).

Patient 1: Colonoscopy revealed non-contiguous and diffuse aphthae throughout the colon (**Figure 2A**). The leopard sign was found slightly in the ascending colon near the ileum (**Figure 2B**). Patient 2: Colonoscopy revealed a diffuse and continuous leopard sign throughout the colon, with slight erythema or erosion (**Figures 2C, D**). Patient 3: Colonoscopy performed before HCT revealed a leopard sign on the colonic mucosa, with slight erythema or erosion (**Figure 2E**).

### 2.2 Histopathological findings

**Figure 3:**
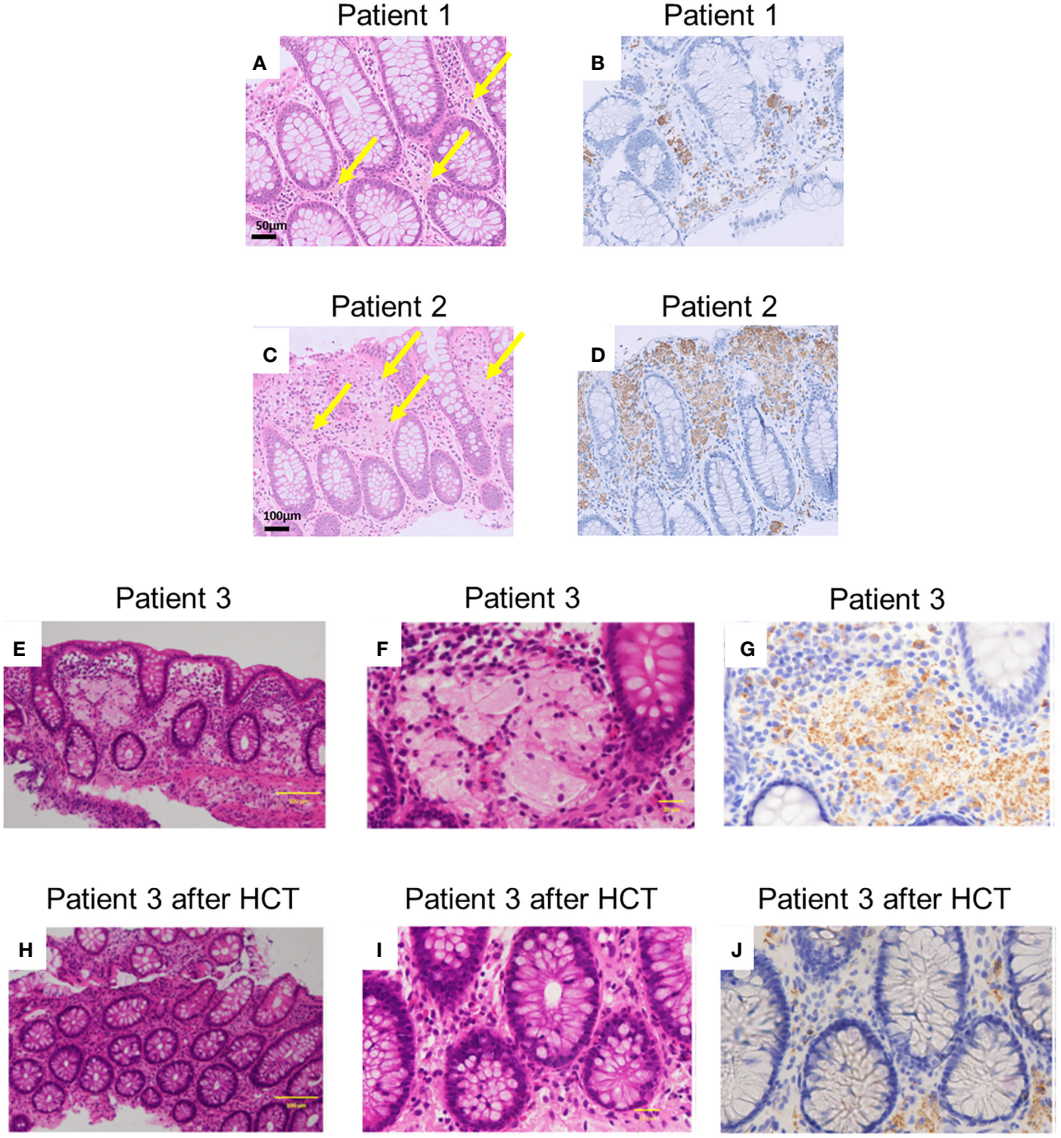
Histopathological findings of patients. **(A, C, E, F, H, I)** depict haematoxylin and eosin staining (**A, C, E**, and **H** are magnified x100, and **F** and **J** are magnified x400). Arrows indicate pigment-laden CD68-positive macrophages (PLMs). **(B, D, G, J)** depict anti-CD68 staining [**(B, D)** are magnified x100, and **(G, J)** are magnified x400]. **(A, B)** Indicate samples from patient 1, and **(C, D)** Indicate samples obtained from patient 2 before prednisolone treatment. **(E-G)** Indicate samples before haematopoietic cell transplantation (HCT) in patient 3. **(H-J)** Indicate the samples after HCT in patient 3. The histological findings revealed aggregates of PLMs in the lamina propria. Patient 1 exhibited fewer PLMs in biopsied specimens compared to patients 2 and 3. Following HCT, patient 3’s biopsied specimens showed a decrease in PLMs compared to those observed before HCT.

The histological findings of patients 1–3 revealed aggregates of pale brown pigment-laden CD68-positive macrophages (PLMs) in the lamina propria (**Figures 3A–G**). In patient 1 (**Figures 3A, B**), biopsied specimens showed fewer PLMs than in patients 2 and 3 (**Figures 3C–G**).

### 2.3 Patient follow-up details

In patient 1, therapy was initiated with oral prednisolone (1 mg/kg) for colitis management. His symptoms disappeared soon after the treatment began. His FCP level decreased to 72 mg/kg two months after the start of treatment. Oral prednisolone doses were tapered and discontinued over the next 3 months. In patient 2, therapy was initiated with oral prednisolone (1 mg/kg) for colitis management. His anal fistula improved, and his weight gain was also noted. However, in the follow-up colonoscopy 2 months after therapy, the brown dots appeared faded, and the yellowish oedematous mucosa was unchanged (**Figure 2F**). Oral prednisolone doses were tapered and discontinued over the next 3 months. Patient 3 underwent HCT with a transplant from his haploidentical father. The conditioning regimen consisted of fludarabine, rabbit anti-thymocyte globulin, targeted busulfan, and total body irradiation at 3 Gy. After HCT, the patient received post-transplant cyclophosphamide on days +3 and +4, and prophylaxis for graft-versus-host disease (GVHD) was administered using mycophenolate mofetil and tacrolimus from day +5 (10, 11). He achieved neutrophil engraftment on day +14, and full donor chimaerism was confirmed on day +31. Furthermore, acute gut GVHD (stage I) occurred on day +14; however, it improved with the adjustment of the tacrolimus dose. In the follow-up colonoscopy on day +43 after HCT, the brown dots appeared to fade, and the yellowish oedematous mucosa was ameliorated (**Figure 2G**). After HCT, biopsies showed aggregates of granular pigmented macrophages, whose population had decreased compared with that before HCT (**Figures 3H–J**). In addition to changes in macrophages, cryptitis was observed in prominent mononuclear cells, neutrophils, and eosinophils. Moreover, apoptosis, which appears to be caused by GVHD of the gut, was also observed.

## 3 Discussion

Colonoscopies of patients with CGD generally show colitis, patchy friability, ulcers, and pseudopolyps, which are frequently observed in patients with ulcerative colitis. In contrast, the histological findings of patients with CGD show that eosinophils are grouped around crypts and are involved in granuloma, cryptitis, and the formation of crypt abscesses resembling Crohn’s disease (4–6). These endoscopic and histological findings appear to be nonspecific, making it difficult to differentiate between CGD and other forms of colitis, such as Crohn’s disease (12). Since certain patients with CGD initially have GI symptoms without any episode of infection, they can be misdiagnosed with Crohn’s disease. Therefore, these patients might be associated with unexpected invasive infections when they do not receive appropriate prophylactic therapy.

Patient 1 presented with GI symptoms that were strongly suggestive of CGD-associated colitis. Patient 2 did not have GI symptoms but had a perianal lesion, which is sometimes observed in patients with Crohn’s disease. The FCP level, which is considered to reflect the disease status of CGD colitis (13), was elevated in patient 1 but not in patient 2. Intriguingly, Patient 3 had no GI or perianal lesions. Therefore, the presence of the leopard sign may not reflect the clinical disease activity in patients with CGD-associated colitis.

We considered that the “leopard sign” serves as a significant endoscopic marker for CGD and may be a characteristic feature of this condition. Whereas it has been reported in other diseases such as celiac disease (14). The characteristics of the “leopard sign” in CGD differ significantly. In cases of CGD, the dot size is larger, and the appearance is distinct, making it distinguishable from other conditions. Notably, the leopard sign has not been reported in other inflammatory bowel diseases (IBD). This is a significant finding as it often presents challenges in distinguishing between IBD-CGD and other IBD cases. Histopathological findings of the leopard sign show large macrophages with brown granular cytoplasm or pink eosinophilic crystalline cytoplasmic inclusions. The NADPH oxidase complex is crucial for protecting phagocytes from microorganisms, such as bacteria and fungi, and is composed of membrane-bound (gp91^phox^ and p22^phox^) and cytosolic proteins (p47^phox^, p67^phox^, p40^phox^, and Rac). This complex generates reactive oxygen species, such as hydrogen peroxide and hydroxyl radicals, which are highly toxic to phagocytosed microorganisms (2). In the absence of NADPH oxidase, caused by CGD-related mutations, the bacteria and their residues cannot be cleared, leading to the formation and accumulation of cytoplasmic pigments. Because pigmented macrophages are not associated with acute inflammation in CGD (4), they might be found in patients without acute inflammation, such as patients 2 and 3. In a large cohort of patients with CGD-associated colitis, the presence of the leopard sign has not been described, although pigmented macrophages were observed in 65% of colon biopsies (4). Leopard sign may be a characteristic endoscopic finding primarily in Japanese patients with CGD.

Patient 1 had ameliorated colitis, although colonoscopy was not performed after prednisolone treatment. Although patient 2 responded to prednisolone treatment, the leopard signs did not change after treatment. In patient 3, the leopard sign faded after HCT. The HCT has restored the activity of NADPH oxidase, which resulted in the normalisation of the function of scavenger macrophages and the elimination of pigment accumulation.

Although X-linked CGD accounted for approximately 70% of CGD cases (1, 2), all patients had X-linked CGD caused by a variant in *CYBB*. Patients with X-linked CGD usually have more severe diseases with earlier presentation than those with autosomal recessive CGD (3). Moreover, it has been reported that the rate of GI involvement is much higher in patients with X-linked CGD than in those with the autosomal recessive form (15). Therefore, the leopard sign may be more frequent and specific in patients with X-linked CGD. Compared to patients 1 and 3, patient 2 had a missense variant at amino acid 182 of the *CYBB* gene, and the results of DHR123 flow cytometry testing suggested that NADPH oxidase activity was retained (**Figure 1**). Therefore, patient 2 may have had an older age of onset and may have had milder clinical symptoms (16, 17). However, even in this patient, the leopard sign was observed in the endoscopy. When the leopard sign is observed on endoscopy in the presence of IBD symptoms, physicians should consider CGD and perform DHR123 testing. Patients with CGD exhibiting the leopard sign and lacking GI symptoms likely do not require specific treatment for CGD-associated colitis.

In conclusion, the leopard sign is not always associated with GI symptoms but is a characteristic endoscopic finding of CGD-associated colitis; however, not all patients show this sign. HCT treatment ameliorated the leopard sign, histopathological findings, and GI symptoms. However, the leopard sign does not reflect the severity of CGD-associated colitis. Overall, our study will promote efficient diagnosis and better management of the patients.

## Data availability statement

The datasets for this article are not publicly available due to concerns regarding participant/patient anonymity. Requests to access the datasets should be directed to the corresponding author.

## Ethics statement

The studies involving humans were approved by The Ethics Committee on Clinical Research of the Sakuragaoka Campus, Kagoshima University. The studies were conducted in accordance with the local legislation and institutional requirements. Written informed consent for participation in this study was provided by the participants’ legal guardians/next of kin. Written informed consent was obtained from the minor(s)’ legal guardian/next of kin for the publication of any potentially identifiable images or data included in this article.

## Author contributions

TN and TT wrote the manuscript. TN, TT, AN, JN, TO, TK, and HK collected patient data. MK, ATani, and SKi evaluated the histopathology. ATana, SKa, and KO performed endoscopy. YO and TM provided critical discussions. HK conceptualised the study and edited the manuscript. All authors contributed to the article and approved the submitted version.
